# Paroxysmal Nocturnal Hemoglobinuria with a Distinct Molecular Signature Diagnosed Ten Years after Allogenic Bone Marrow Transplantation for Acute Myeloid Leukemia

**DOI:** 10.1155/2019/8928623

**Published:** 2019-02-05

**Authors:** Alberto Santagostino, Laura Lombardi, Gerard Dine, Pierre Hirsch, Srimanta Chandra Misra

**Affiliations:** ^1^Department of Clinical Hematology, Troyes General Hospital, 10000 Troyes, France; ^2^Sorbonne University, Inserm, Centre de Recherche Saint-Antoine, CRSA, AP-HP, Department of Laboratory Hematology Saint-Antoine Hospital, Paris, France; ^3^Department of Clinical Hematology, Saint Antoine Hospital, Paris, France

## Abstract

Paroxysmal nocturnal hemoglobinurea (PNH) is a rare disorder of complement regulation due to somatic mutation of PIGA (phosphatidylinositol glycan anchor) gene. We herewith report a case who developed a symptomatic PNH long after an allogenic marrow transplant. Some reasonable arguments concerning the origin of PNH clone have been discussed. The molecular studies revealed presence of JAK2 and TET2 mutations without a BCOR mutation. The literature review has been performed to probe into the complex interplay of autoimmunity and clonal selection and expansion of PNH cells, which occurs early in hematopoietic differentiation. The consequent events such as hypoplastic and/or hemato-oncologic features could further be explained on the basis of next-generation sequencing (NGS) studies. Paroxysmal nocturnal hemoglobinuria (PNH) is a rare clonal disorder of hematopoietic stem cells, characterized by a somatic mutation of the phosphatidylinositol glycan-class A (PIGA). The PIGA gene products are crucial for biosynthesis of glycosylphosphatidylinositol (GPI) anchors, which attaches a number of proteins to the plasma membrane of the cell. Amongst these proteins, the CD55 and CD59 are complement regulatory proteins. The CD55 inhibits C3 convertase whereas the CD59 blocks the membrane attack complex (MAC) by inhibiting the incorporation of C9 to MAC. The loss of complement regulatory protein renders the red cell susceptible to complement-mediated lysis leading to intravascular and extravascular hemolysis. The intravascular hemolysis explains most of the morbid clinical manifestations of the disease. The clinical features of syndrome of PNH are recurrent hemolytic episodes, thrombosis, smooth muscle dystonia, and bone marrow failure; other important complications include renal failure, myelodysplastic syndrome (MDS), and acute myeloid leukemia (AML). The most used therapies were blood transfusions, immunosuppressive, and steroid. Allogeneic stem cell transplantation was also practiced. At present, the therapy of choice is eculizumab (Soliris, Alexion Pharmaceuticals), a humanized monoclonal antibody that blocks activation of the terminal complement at C5. The limiting factor for this therapy is breakthrough hemolysis and the frequent dosing schedule. Ravulizumab (ALXN1210) is the second generation terminal compliment inhibitor which seems to provide a sustained control of hemolysis without breakthrough hemolysis and with a longer dosing interval.

## 1. Case Report

A 63-year-old man presented in August 2017 with moderate pancytopenia associated with hemolysis [1]. The blood results were Hb 8.5 g/dL, MCV 103 fL, WBC 3.2 × 109/L, platelets 128 × 109/L, reticulocytes 321 × 109/L, LDH 3462 U/L, reduced haptoglobin <0.01 *μ*mol/L, ferritin 461 ng/mL, total bilirubin 4 mg/dL, creatinine 142 g/L, and normal value of folate and vitamin B12. Clinically, he presented fatigue and cholecystitis.

The patient was diagnosed in 2007 as myelodysplastic syndrome with excess blasts (MDS-EB) followed in another centre. In 2009, the patient evolved acute myeloid leukemia (AML) treated with classic induction therapy idarubicin and cytarabine and consolidation therapy followed by allogeneic stem cells transplantation in 2010 with matched unrelated donor (MUD) achieving a complete remission with complete donor chimerism. In November 2016, he presented a moderate anemia (Hb 10 g/dL), treated with darbepoetin alfa. The aspiration was impossible. The bone marrow biopsy specimen was normocellular with dysmyelopoiesis but without blasts. The annual follow-up at the allotransplant centre showed chimerism of donor as 22% in July 2017. In August 2017, the flow cytometric analysis of peripheral blood cells revealed 96.2% of PNH clone type III. Another control of bone marrow biopsy specimen in September 2017 showed erythroblastic hyperplasia without blasts. Therefore, the diagnosis was paroxysmal nocturnal hemoglobinuria (PNH). The patient began therapy with eculizumab, and prior to this treatment, he required 3 transfusions of packed RBCs. The patient was vaccinated against *Neisseria meningitidis* 2 weeks before the start of treatment. Eculizumab therapy began in October 2017 with an induction dose of 600 mg × 2 intravenous (iv) weekly for 4 weeks followed by a single dose of 900 mg (iv) after 7 days, then 900 mg iv every 15 days. Seven months later, the patient continues eculizumab without bleeding or thrombosis signs and with a stable value of hemoglobin (9-10 g/l). He presents a reduction of hemolysis index and a good quality of life. In molecular analysis, we found JAK2 V617 F mutation with an allelic frequency of 44%, and the NGS study revealed a frameshift mutation of TET2 with an allelic frequency of 34%. The patient is very closely followed up for an imminent relapse. During preparation of this manuscript, the patient relapsed with a diagnosis of AML.

## 2. Discussion

PNH is a rare hemolytic anemia first described in 1882 by Strübing [2] This is a disorder of complement regulation, caused by somatic mutations in the PIGA gene which is 17 kb long with 6 exons and maps to short arm of X chromosome.

The other important disorders of complement regulations are atypical hemolytic uremic syndrome (aHUS), caused by cell surface alternative pathway dysregulation and C3 glomerulopathy (C3G) due to fluid-phase alternative pathway dysregulation The mechanism underlying these dysregulations are diverse, predominantly acquired autoimmune in C3G, somatic mutations in PNH, or an inherited germline mutations in aHUS [3].

The location of PIGA gene in X chromosome explains the ability of the mutation to cause PNH as only one allele is functional in male as well as in female.

The development of PNH involves a multistep process, such as clonal selection and clonal expansion resulting in hypoplastic/aplastic anemia (AA) and sometimes malignant transformation leading to MDS and AML.

Nafa et al. identified 15 different somatic mutations in 12 patients, out of which 10 were caused by frameshift mutations; they postulated that the predominance of frameshift mutation might pave the way for a clonal selection [4].

The clonal selection of PIGA-mutated hematopoietic cells might be immune mediated as PNH has its close association with aplastic anemia (AA). AA may develop during the course of PNH and vice versa (AA/PNH syndrome), both being predisposed to AML and MDS [5].

The complication of clinically relevant PNH has been described in 15% to 25% of the patients with AA treated by immunosuppressive therapy (IST). The clonal hematopoiesis is much higher among patients with aplastic anemia, with high incidence of BCOR and BCORL1 and PIGA mutations, whereas TET2 and JAK2 were infrequently mutated, suggesting a distinct mechanism of clonal selection and thus a complex interplay between immunology and oncogenesis [6]. In our patient, the presence of TET2 and JAK2 without the presence of BCOR is consistent with the fact that the patient never had hypoplastic features. The pathogenesis was driven more towards oncogenetic events.

Moreover, regardless of typical manifestations of PNH, GPI anchor-deficient, “PNH-type” cells are detectable at higher frequencies [7].

There is growing evidence that the expansion of the PNH clone results from T-cell-mediated autoimmune damage to hematopoietic stem cells, with the GPI molecule as target. Indeed, GPI-specific CD8 + T cells, which have been identified in PNH patients, would spare selectively GPI-negative stem cells [8].

However, the mutant PIGA cell clone itself fails to explain a clonal expansion. Hence, there might be a second somatic mutation (second hit) conferring the mutant PIGA cell a proliferative advantage. Inoue et al. [9] were successful in discovering ectopic expression of HMGA2 in the mutant PIGA cell accounting for clonal hematopoiesis.

Later, the deep sequencing by Shen et al. in PNH patients revealed (TET2) zinc finger SUZ12, transcription factor ASXL1, BCL6 corepressor (BCOR), JAK2, and U2 small nuclear RNA auxiliary factor 1 (U2AF1), thus adding the complexity of clonal architecture and growth advantage. They presented data from whole exome sequencing (WES) of clonal (GPI-deficient) and nonclonal cells from PNH patients to examine the mutational history of PNH which depicted 4 different scenarios:

1. PIGA as the initial ancestral event accompanied by secondary mutations
2. PIGA as an event secondary to other mutations
3. PIGA as the lone mutation
4. PIGA mutation coexisting with other mutations responsible for the development of an MDS clone

However, in some cases, the leukemogenic effects might be driven by mutations such as JAK2, TET2, or STAC3, suggesting that these primary events arose, conveying an initial growth advantage, with a PIGA mutation as a subclone conveying an additional growth advantage [10]. The STAC3 mutation was not studied in our patient; Mochizuki et al. [11] supported the suggestion that GPI-deficient stem cells have a survival advantage in the setting of immune-mediated BM injury or postallotransplant.

We do not know the origin of the PNH clone (donor or patient). This is an open question. We postulate the PNH clone was derived from the patient because our patient presents a reduction of donor chimerism of 22% at the PNH debut but the analysis of donor PNH clone was not performed. In our patient, there are two hypotheses which merit a serious consideration.

Firstly, whether the clonal selection of PIGA-mutated clone is driven by donor T cell irrespective of the origin of the clone. The possibility is quite low as the PNH clone emerged following reduction of chimerism and before the relapse of MDS.

The second possibility is that the patient might be harboring a coexistence of MDS and PNH clone long before the diagnosis of SMD. This second hypothesis might be appropriate in our case.

Some intrinsic immune dysregulation led a clonal selection of both of the clones, which obtained proliferative advantage. This hypothesis is further consolidated by the findings of the high frequency of the driver mutation JAK2 as well as TET2 mutation. In 2007, JAK2 was not systematically searched for, and the implication of TET2 was unknown before 2009.

Hence, the AML diagnosed in 2007 probably arose from a preexisting myeloproliferative/myelodysplastic overlapping syndrome. The present PNH clone was probably driven by the existing MPN/MDS. This intriguing issue questioned long back by Professor Willium Dameshek in 1969 who made a proposal to consider PNH as a candidate disorder of myeloproliferative neoplasm (MPN) [12]. The definite classification of MDS came only in 1982 as an attempt to define different preleukemic conditions, where as in 1950s, Professor Damashek had already made a conceptual construct of MPN.

We conclude that the PNH clone belongs to the receiver which reappeared during loss of chimerism and parallels with the relapse.
